# Evaluating the Cellular Targets of Anti-4-1BB Agonist Antibody during Immunotherapy of a Pre-Established Tumor in Mice

**DOI:** 10.1371/journal.pone.0011003

**Published:** 2010-06-08

**Authors:** Gloria H. Y. Lin, Yuanqing Liu, Thanuja Ambagala, Byoung S. Kwon, Pamela S. Ohashi, Tania H. Watts

**Affiliations:** 1 Department of Immunology, University of Toronto, Toronto, Ontario, Canada; 2 Cell and Immunobiology and R&D Center for Cancer Therapeutics, National Cancer Center, Ilsan, Korea; 3 The Campbell Family Cancer Research Institute at Princess Margaret Hospital, Toronto, Ontario, Canada; 4 Department of Medicine, Tulane University, New Orleans, Louisiana, United States of America; University of Toronto, Canada

## Abstract

**Background:**

Manipulation of the immune system represents a promising avenue for cancer therapy. Rational advances in immunotherapy of cancer will require an understanding of the precise correlates of protection. Agonistic antibodies against the tumor necrosis factor receptor family member 4-1BB are emerging as a promising tool in cancer therapy, with evidence that these antibodies expand both T cells as well as innate immune cells. Depletion studies have suggested that several cell types can play a role in these immunotherapeutic regimens, but do not reveal which cells must directly receive the 4-1BB signals for effective therapy.

**Methodology/Principal Findings:**

We show that re-activated memory T cells are superior to resting memory T cells in control of an 8-day pre-established E.G7 tumor in mice. We find that *ex vivo* activation of the memory T cells allows the activated effectors to continue to divide and enter the tumor, regardless of antigen-specificity; however, only antigen-specific reactivated memory T cells show any efficacy in tumor control. When agonistic anti-4-1BB antibody is combined with this optimized adoptive T cell therapy, 80% of mice survive and are fully protected from tumor rechallenge. Using 4-1BB-deficient mice and mixed bone marrow chimeras, we find that it is sufficient to have 4-1BB only on the endogenous host αβ T cells or only on the transferred T cells for the effects of anti-4-1BB to be realized. Conversely, although multiple immune cell types express 4-1BB and both T cells and APC expand during anti-4-1BB therapy, 4-1BB on cells other than αβ T cells is neither necessary nor sufficient for the effect of anti-4-1BB in this adoptive immunotherapy model.

**Conclusions/Significance:**

This study establishes αβ T cells rather than innate immune cells as the critical target in anti-4-1BB therapy of a pre-established tumor. The study also demonstrates that *ex vivo* activation of memory T cells prior to infusion allows antigen-specific tumor control without the need for reactivation of the memory T cells in the tumor.

## Introduction

Despite extensive evidence that CD8 T lymphocytes can recognize and kill cancer cells, malignant tumors are rarely controlled by spontaneous immune responses [1]. Thus there is great interest in manipulating CD8 T cells to enhance their ability to seek out and kill tumor cells. Adoptive T cell therapy, in which autologous cells from the patient are expanded *ex vivo* and reintroduced into the patient, represents a promising approach for activating the immune response against cancer [1], [2]. However, further optimization of these approaches will require an understanding of the cell types and mechanisms required for tumor control in an immunotherapeutic context.

One approach to enhancing CD8 T cell-based cancer therapy is to use immune modulators targeting T cell survival and effector pathways. The TNFR family member 4-1BB is a potent survival factor for activated and memory CD8 T cells [3]–[9]. 4-1BB is superior to CD28 in expanding T cells for adoptive therapy [10] and 4-1BBL-expanded CD8 T cells have increased effector function per cell [10], [11]. Thus 4-1BB agonists represent attractive candidates for combination therapy with adoptively transferred CD8 T cells. Since the initial observation that agonistic anti-4-1BB antibodies promote tumor regression in mice [12], a large number of studies have shown efficacy of 4-1BB stimulation in anti-cancer therapies (Reviewed in [13], [14]). Indeed phase I trials are underway using humanized anti-4-1BB agonist antibodies for advanced cancers (reviewed in [14]). To further improve these therapies in a rational way, it will be important to understand the cellular targets involved in the response to anti-4-1BB therapy [15].

Another key issue for optimization of adoptive T cell therapy has been to determine the most efficacious T cell subset for the eradication of tumors *in vivo*. CD8 T cells can display an “effector”, “effector memory”, or a “central memory” phenotype [16]. A recent report suggests that reactivation of effector memory T cells prior to adoptive transfer removes the need for additional immunization during adoptive T cell therapy, resulting in effective tumor control following *in vitro* programming of the T cells [17]. Whereas primary effector or effector memory CD8 T cells are superior in target killing, central memory CD8 T cells have a survival advantage [16]. CD8 T cells expanded in IL-15 have a survival advantage over IL-2 generated CD8 effector T cells [18] and IL-15 induced central memory cells show more effective tumor control than IL-2 generated effector T cells [19]–[21]. Consistent with this hypothesis, persistence of transferred T cells correlates with cancer regression in an adoptive T cell therapy trial of metastatic melanoma [22]. As effector cells reactivated from central memory T cells show more persistence than effectors obtained from effector memory T cells after transfer into macaques [23], these cells appear to be the preferred subset for adoptive therapy.

Here we demonstrate that effector T cells reactivated from central memory T cells (“reactivated memory”) are indeed more efficacious in tumor control than resting central memory T cells (“resting memory”) against an established tumor. We then took this optimized T cell therapy model and combined it with *in vivo* treatment with agonistic anti-4-1BB antibodies. T cells reactivated from ovalbumin-specific OT-I memory T cells together with anti-4-1BB agonistic antibody resulted in 80% tumor free survival in mice bearing 6–9 mm pre-established tumors. We next went on to determine the mechanism of action of 4-1BB in this model.

Cell depletion studies have implicated a number of cell types in tumor therapy with anti-4-1BB [12], [24]–[29]. However, while such studies indicate which cells in the host are required for tumor control, they do not reveal the direct therapeutic targets of anti-4-1BB. Systemic administration of anti-4-1BB agonistic antibody at doses of as little as 200 µg/mouse has been shown to cause splenomegaly and other immune system anomalies [30], therefore it is essential to determine the important targets of anti-4-1BB for tumor control so that more focused therapies can be devised to potentially avoid immunotoxicity. In this report we used 4-1BB-deficient mice and bone marrow chimeras to address this issue. Despite the efficacy of anti-4-1BB in expanding innate immune cells, as well as host T cells, we found that 4-1BB on the transferred T cells or on the host αβ T cells is necessary and sufficient for the effects of anti-4-1BB on adoptive tumor therapy against the E.G7 thymoma. Conversely, expression of 4-1BB on cells other than αβ T cells is neither necessary nor sufficient for the effect of 4-1BB agonists even when the transferred T cells lack 4-1BB. These studies pinpoint T cells rather than innate immune subsets as the major target of 4-1BB agonists in cancer therapy against this rapidly growing thymoma.

## Results

### Reactivated memory CD8 T cells manifest superior anti-tumor activity compared to resting memory T cells

**Figure 1:**
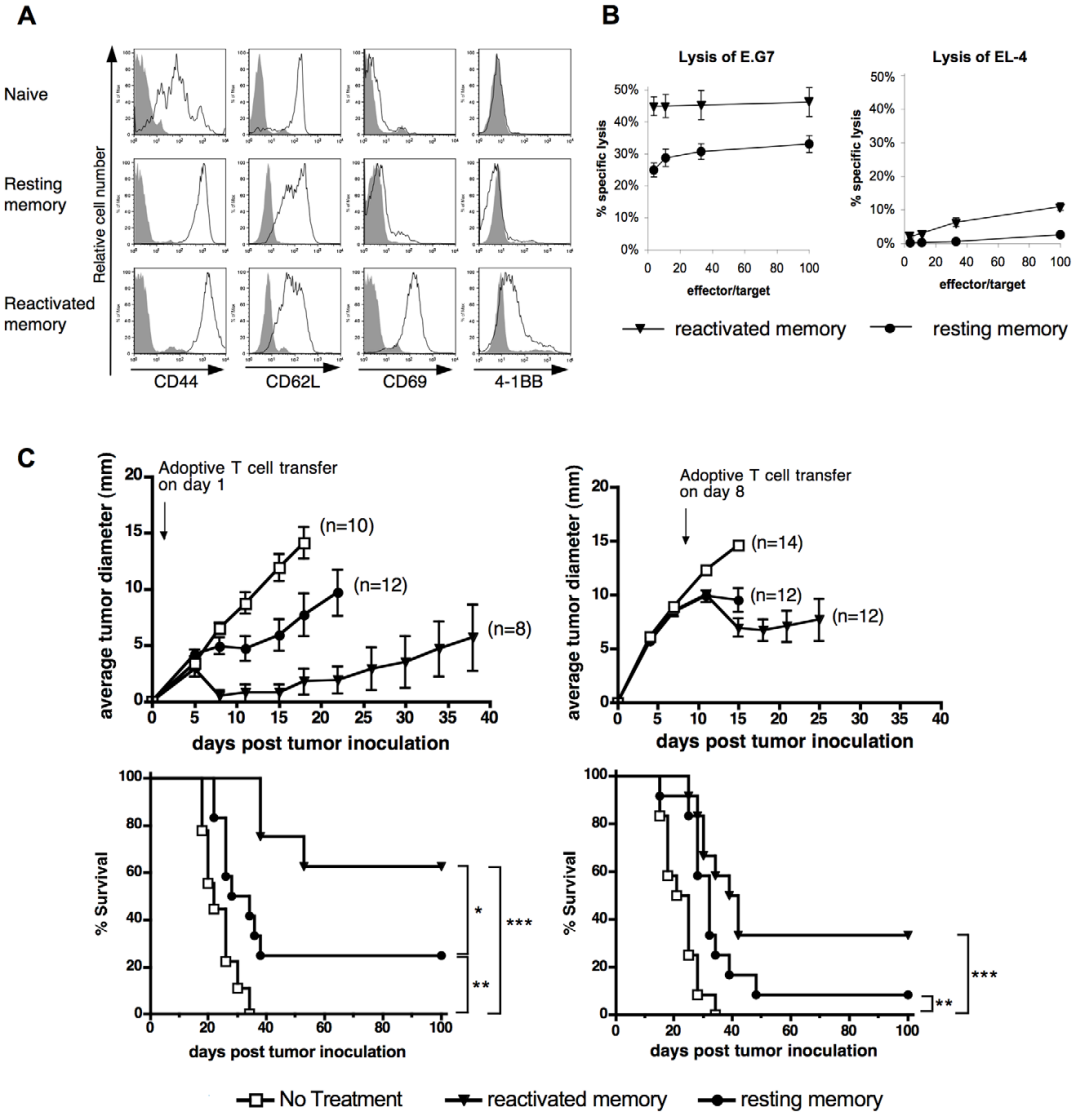
Superior anti-tumor activity of reactivated compared to resting memory T cells. (A) Phenotypes of naïve, *in vitro* generated resting and reactivated OT-I memory cells. OT-I cells were surface stained for the expression of CD44, CD62L, CD69 and 4-1BB (open areas), overlaid on the background staining with isotype controls (shaded areas). Histograms were gated on the CD8^+^ population. (B) *In vitro* killing of E.G7 target cells by resting or reactivated memory OT-I cells. The cytotoxicity of resting and reactivated memory OT-I cells was tested on day 9 of culture, in a 4-hour Cr^51^ release assay at the indicated effector to target ratios. Parental (ovalbumin negative) EL-4 cells were used as a negative control. (C) Adoptive immunotherapy of E.G7 tumor bearing animals with reactivated or resting memory OT-I cells. Mice were inoculated s.c. with E.G7 cells on day 0. One day (left panel) or eight days (right panel) later, tumor-bearing mice received 3×10^6^ OT-I resting memory or reactivated memory cells i.v. or no cell transfer, as indicated. Average tumor growth, the number (n) of mice per group and survival of tumor bearing mice are shown. Tumor growth curves were discontinued at the time when the first animal in the group succumbed to the disease. Results are pooled from two independent experiments. Survival curves were analyzed by the LogRank test, with *P<0.05, **P<0.01 and ***P<0.001.

To test the role of 4-1BB in an adoptive therapy model, we first set out to optimize the adoptive therapy using *in vitro* generated memory T cells. To generate a large number of Ag-experienced OT-I T cells *in vitro*, OT-I splenocytes were cultured with peptide Ag followed by IL-15, resulting in a 20- to 30-fold expansion of the T cells by day 9 (data not shown). OT-I T cells generated in IL-15 displayed a resting “central memory” phenotype as indicated by their high surface expression of CD44 and CD62L with low to undetectable CD69 and 4-1BB expression (Fig. 1A). Reactivated memory T cells were generated by stimulating the IL-15-expanded memory T cells with irradiated peptide-loaded syngeneic splenocytes for 2 days prior to transfer. Reactivated memory T cells markedly up-regulated 4-1BB and CD69 expression, while modestly down-regulating CD62L (Fig. 1A). The reactivated memory T cells exhibited a 50–80% greater cytotoxicity against E.G7 targets than the resting memory T cells (Fig. 1B). It should be noted that the rapid reactivation of effector function of these *in vitro* generated “central memory” cells, supports the contention that they have acquired the properties of memory cells, as restimulation of primary effectors results in activation induced cell death [31], [32].

To test the efficacy of resting versus reactivated memory T cells *in vivo*, three million OT-I cells were injected intravenously (i.v.) into mice bearing E.G7 tumors. This cell number/body weight ratio was relevant to clinical doses for adoptive cancer therapy [33]. Animals were inoculated with E.G7 tumor cells either one day (Fig. 1C left panel) or 8 days before T cell transfer (Fig. 1C right panel). A subcutaneous E.G7 tumor grew rapidly in untreated C57BL/6 mice, reaching 6–9 mm in diameter by one week after inoculation. Untreated animals succumbed to a large progressive tumor (+/− 20×20 mm) in 2–4 weeks. When OT-I T cells were administered, a delay in tumor growth was observed. Reactivated memory T cells were more potent than resting memory cells based on adoptive transfer of the same number of T cells in both the 1-day tumor and 8-day tumor settings, although the effect was more pronounced in the small tumor setting. The fate of the tumor bearing animals was followed for up to 100 days. Using reactivated memory T cells, 63% or 33% of animals were tumor-free in the small or large tumor setting, respectively. When resting memory cells were injected, only 25% of mice in the small tumor group and 8% in the large tumor group were tumor free by day 100. Thus, reactivated memory CD8 T cells were more potent than resting memory cells in this model of adoptive T cell therapy. Subsequent experiments were performed using the more stringent 8-day tumor setting.

**Figure 2:**
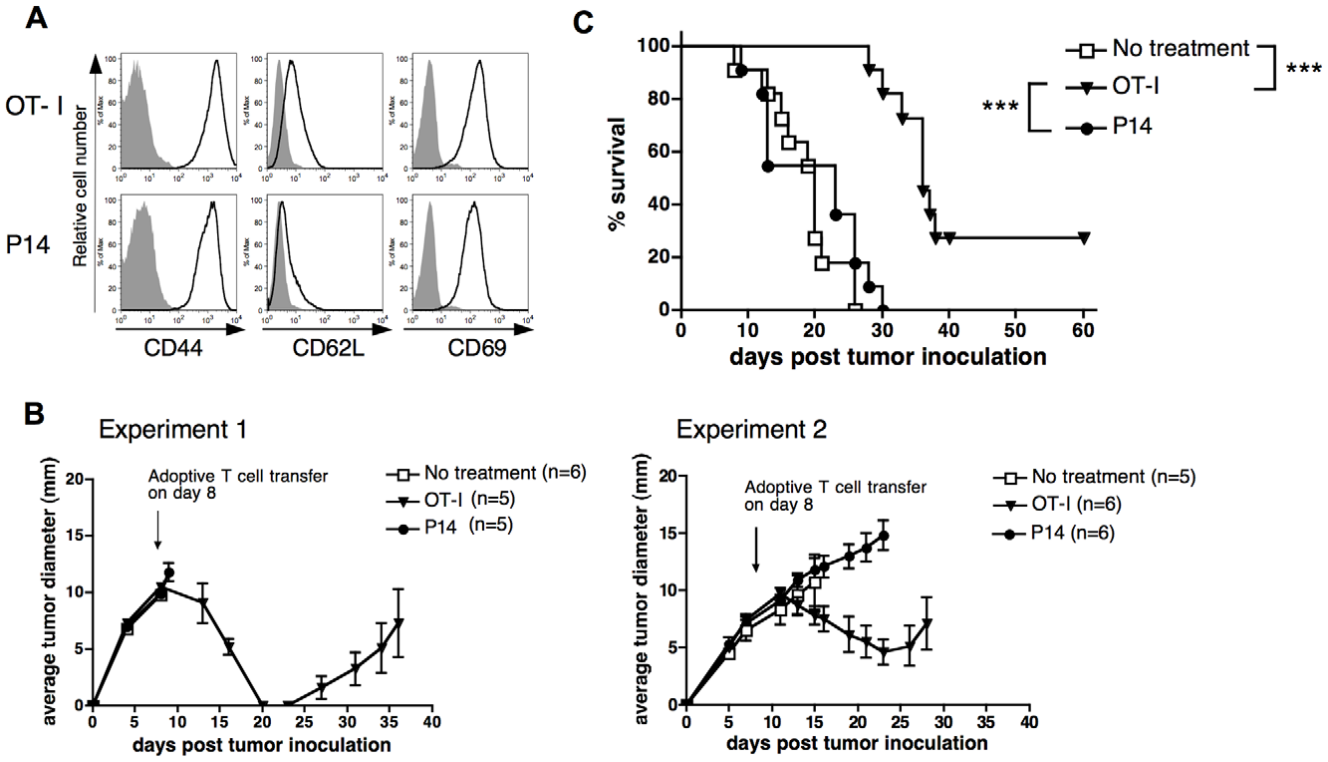
Antigen specificity of tumor control by reactivated memory T cells. (A) Phenotypes of *in vitro* generated reactivated OT-I and P14 memory cells. T cells were surface stained for the expression of CD44, CD62L and CD69 (open areas) overlaid on the background staining with isotype controls (shaded areas). Histograms were gated on the CD8^+^ population. (B) Mice bearing eight-day E.G7 tumors were given 3×10^6^ reactivated OT-I or P14 memory cells i.v. or no cell transfer, as indicated. Average tumor growth is shown for two independent experiments, with (n) indicating number of mice per group. Tumor growth curves were discontinued at the time when the first animal in the group succumbed to the disease. (C) Survival data for tumor bearing mice were pooled from two independent experiments. Survival curves were analyzed by the LogRank test, with ***P<0.001.

It was possible that the reactivated effectors were more potent than resting memory T cells in controlling the tumor due to the effect of transferring pre-activated T cells which might result in Ag-non-specific tumor regression due to their cytokine production. Therefore, in a parallel set of experiments we compared reactivated memory T cells from OT-I mice with those obtained from P14 TCR transgenic mice, specific for LCMV glycoprotein gp_33–41_ [34]. Reactivated memory OT-I and P14 T cells showed a similar surface phenotype (Fig. 2A). However, only the reactivated OT-I memory T cells showed any efficacy in tumor control (Fig. 2B and C). Thus reactivated memory T cells control tumor growth in an antigen-specific fashion.

### Analysis of adoptively transferred resting and reactivated memory CD8 T cells in tumor bearing animals

**Figure 3:**
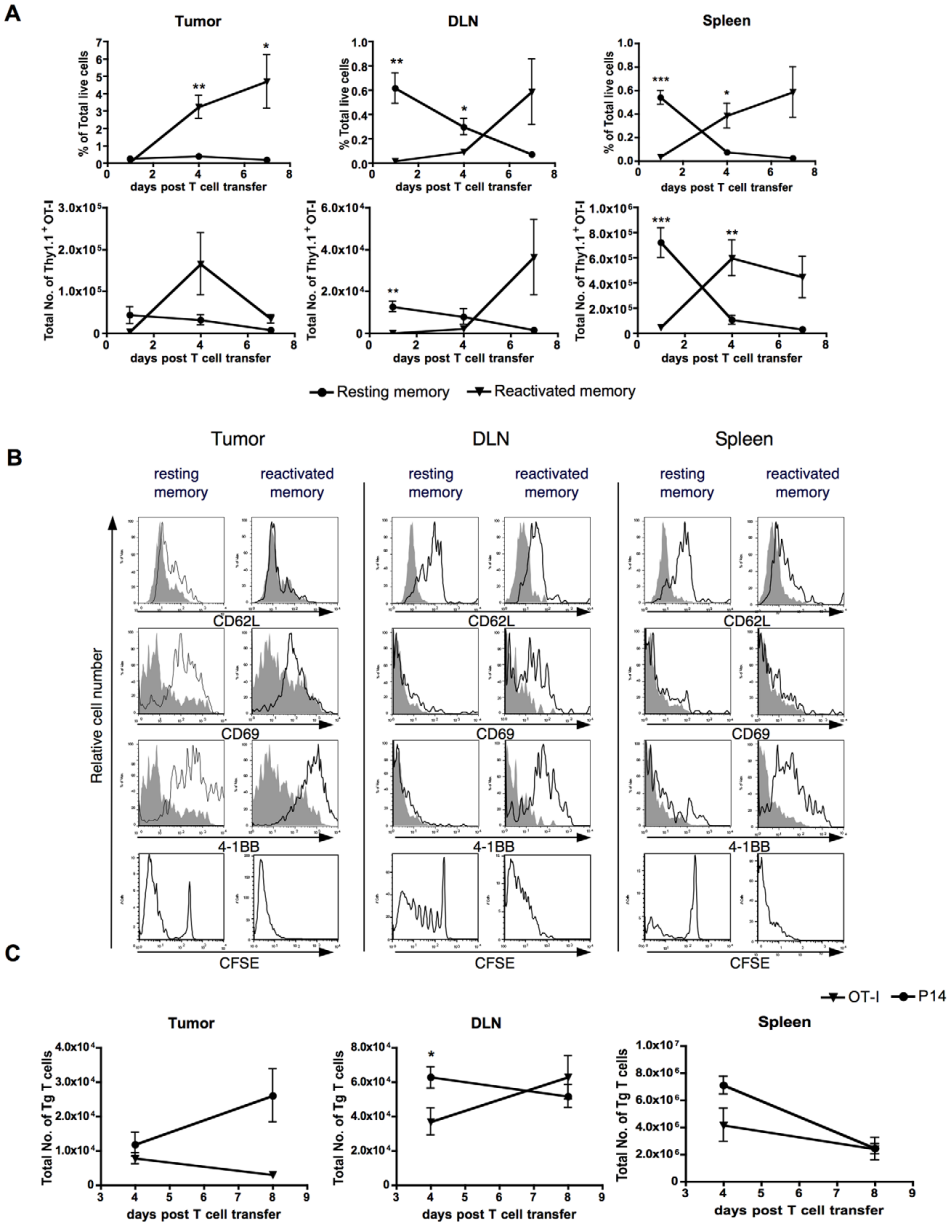
Analysis of memory OT-I cells after adoptive transfer into tumor bearing animals. C57BL/6 mice were inoculated s.c. with E.G7 tumor cells on day 0. On day 8, resting memory or reactivated memory OT-I cells were transferred into tumor bearing mice. On day 1, 4, or 7, mice were sacrificed and OT-I Thy1.1 cells were enumerated in the tumor, draining lymph node (DLN) and spleen. (A) The percentage of OT-I resting memory and reactivated memory cells was determined according to gated CD8^+^ Thy1.1^+^ cells over the total number of living cells. The OT-I cell number was obtained by multiplying the percentage by the total cell count of the organ. A summary of two independent experiments is shown. (B) Histograms show the expression of CD62L, CD69 and 4-1BB (open area) overlaid on the background staining with antibody isotype controls (shaded area) on *ex vivo* resting memory and reactivated memory OT-I Thy1.1^+^ cells in tumor bearing mice 4 days after adoptive transfer. The lower panel shows the proliferation of CFSE labeled OT-I Thy1.1 cells four days after transfer. Similar results were obtained from two independent experiments with 3 mice per group. (C) Reactivated OT-I or P14 memory cells were labeled with CFSE and adoptively transferred to 8 day-tumor bearing mice as in Figure 2B, C. Recovery of the reactivated memory cells was analyzed on day 4 and 8 following adoptive transfer. Day 4 results are representative of two experiments, and the day 8 results were obtained in one experiment, with 2–5 mice per group per time point. By day 4, there was a complete absence of CFSE signal for both OT-I and P14 cells in all organs, indicating extensive division (data not shown). Results in A and C were analyzed by unpaired t-test at each individual time point, with *P<0.05, **P<0.01, and ***P<0.001.

We next examined the primary tumor, draining lymph node and spleen for the congenically marked Thy1.1 OT-I T cells at day one, four and seven after transfer (Fig. 3A, B). Distinct *in vivo* kinetic patterns were noted for resting memory and reactivated memory OT-I cells. As shown in figure 3A, 0.72±0.12×10^6^ Thy1.1 positive cells were recovered in the spleen, constituting about 25% of resting memory T cells that were transferred. In contrast, reactivated memory cells were nearly undetectable (0.04±0.004×10^6^ in the spleen) at this time point (1 day post-transfer). However, a remarkable increase of Thy1.1 positive cells was observed 3 days later in the tumor, DLN and spleen of mice that received reactivated memory T cells. The increased OT-I cell accumulation was most striking in the tumor, in which a >100-fold increase in percentage and >30-fold in total number of Thy1.1 positive cells were recorded. The rapid cell division by reactivated memory OT-I cells following adoptive transfer was further confirmed by transferring CFSE-labeled T cells (Fig. 3B). Active expansion was evident for the reactivated memory OT-I cells between one to seven days following adoptive transfer, as reflected by an increased frequency of Thy1.1 cells in the regressing primary tumors, DLN and spleen, as well as an increased/sustained total number of Thy1.1 cells in the DLN and spleen (Fig. 3A). Concomitantly, tumor regression occurred in the majority of animals treated with reactivated memory T cells (Fig. 1C). In contrast, despite their initial high recovery on day 1 the adoptively transferred resting memory T cells expanded poorly in the spleen and DLN and their percentage and number declined in all the tissues/organs examined (Fig. 3A, B).

We examined the adoptively transferred cells for evidence of activation by measuring surface markers of activation on Thy1.1 positive cells. Whether the mice had received resting or reactivated memory cells, the OT-I cells recovered from the tumor showed an activated T cell phenotype with low CD62L expression and high levels of CD69 and 4-1BB (Fig. 3B). Both T cell populations in the tumor also displayed IFN-γ producing capability (data not shown). Thus, cells that have entered the tumor have an activated phenotype regardless of their starting phenotype.

Analysis of activation markers as well as CFSE staining of transferred cells in the DLN and spleen indicated that the resting memory cells were less activated and had undergone fewer divisions than the effector cells derived by reactivation of memory T cells. In general, there was less activation and less evidence of division in the DLN and spleen than in the tumor for both kinds of transferred T cell (Fig. 3B).

The above studies show that reactivated memory T cells divide substantially more than resting memory T cells after transfer into the tumor-bearing host in both the tumor as well as in the secondary lymphoid organs. Previous studies have shown that CD8 T cells exposed to antigen *ex vivo* for a brief period are committed to undergo several rounds of division in the absence of the continuous presence of antigen [35]–[37]. Thus the expansion of reactivated memory T cells *in vivo* could be due to continued division after stimulation *ex vivo*, or due to re-encounter with Ag *in vivo*. To distinguish these possibilities, we conducted a similar experiment comparing CFSE-labeled reactivated P14 or OT-I memory T cells. By day 4, both P14 and OT-I T cells in all organs were CFSE negative (data not shown), indicating that they had undergone extensive division. However, by day 8, there were fewer transferred T cells found in the tumor of mice that had received OT-I compared to those that had received P14 T cells (Fig. 3C), likely reflecting that the OT-I T cells have already started to destroy the tumor by this point, whereas with the P14 transferred mice, the T cells have no effect on tumor growth (Fig. 2B). Thus, the ability to enter the tumor and continue to divide appears to be a property of the *ex vivo* activation of the T cells rather than a result of the T cells specifically encountering antigen *in vivo* and tumor infiltration of activated T cells does not necessarily correlate with protection. This result suggests that, even in the relatively immunogenic E.G7 model, the tumor microenvironment is still unfavorable or suppressive for optimal activation of adoptively transferred memory T cells. The use of *ex vivo* reactivated memory T cells allows the T cells to expand in vivo, enter and eradicate the tumor, thereby overcoming the problem of needing to reactivate the memory T cells within the tumor.

### Effect of agonistic anti-4-1BB on adoptive therapy with reactivated memory T cells

Although reactivated memory OT-I cells were more effective in treating an established cancer as compared to resting memory OT-I cells, in this stringent 8-day E.G7 tumor model only about 30% of tumor bearing mice remained tumor-free for 100 days after tumor onset (Fig. 1). Therefore, it was of interest to determine whether the response obtained with reactivated memory T cells could be improved by a combined therapy with anti-4-1BB. In addition to showing better tumor control, the reactivated effectors also express higher levels of 4-1BB than the resting memory T cells (Fig. 3B) and therefore should be more responsive to anti-4-1BB therapy.

**Figure 4:**
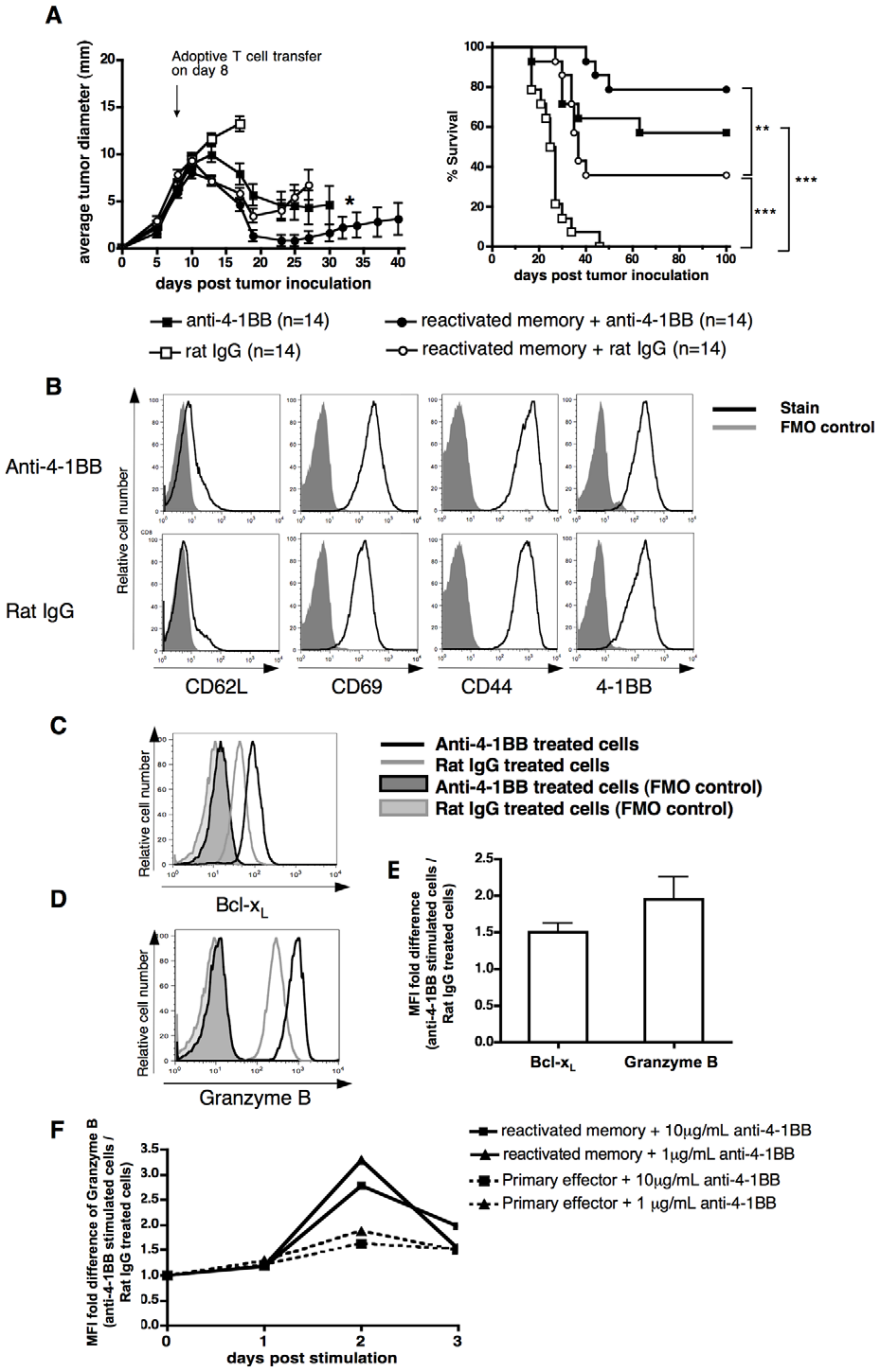
Combination immunotherapy with agonistic anti-4-1BB antibody and reactivated memory CD8 T cells. (A) C57BL/6 mice were inoculated s.c. with E.G7 tumor cells on day 0. On day 8, adoptive transfer was performed by injecting 3×10^6^ reactivated memory OT-I cells i.v. into tumor bearing mice. The agonistic anti-4-1BB antibody (3H3, 100 µg per mouse) was administrated by i.p. injection immediately following T cell adoptive transfer and repeated once 3 days later. Average tumor growth, the number (n) of mice per group and survival of tumor bearing mice are shown. Results are pooled from two independent experiments. (* Tumor growth curve discontinued at the time when the first animal in the group succumbed to the disease, except for the anti-4-1BB antibody treated group in which one tumor bearer was sacrificed on day 18). Survival curves were analyzed by the LogRank test, with **P<0.01 and ***P<0.001. (B–E) Resting memory T cells, prepared as in Figure 1, were reactivated with peptide in the presence of 10 µg/mL of rat IgG or anti-4-1BB agonist for two days. Expression levels of the activation markers (B), Bcl-x_L_ (C) and granzyme B (D) were analyzed. Representative histograms from one of the two independent experiments are shown. (E) Summary of the enhancement of Bcl-x_L_ and granzyme B from two experiments. Median fluorescence intensity (MFI) fold differences are calculated as follows: [(MFI of the staining of anti-4-1BB stimulated cells/MFI of the FMO of anti-4-1BB stimulated cells)]/[(MFI of the stain of the rat IgG treated cells/MFI of the FMO of the rat IgG treated cells)]. (F) Resting memory OT-I T cells, in parallel with purified naïve OT-I CD8 T cells, were activated with peptide pulsed splenocytes in the presence of 1 µg/mL or 10 µg/mL of rat IgG or anti-4-1BB agonist. Kinetics of the induction of granzyme B is shown. Median fluorescence intensity (MFI) fold differences were calculated as in figure 4E.

We chose a dose of 100 µg/mouse of anti-4-1BB delivered at day 0 and 3 after T cell transfer, based on previous studies showing efficacy of anti-4-1BB at this dose [12]. Administration of agonistic anti-4-1BB antibody alone induced complete tumor regression in 57% of animals while all animals in the control group succumbed to a progressive cancer within 50 days (Fig. 4A). When the anti-4-1BB antibody was used together with adoptive transfer of reactivated memory T cells, tumor rejection was achieved in 79% of animals as compared to a 36% tumor rejection rate in mice that received only adoptive T cell transfer (Fig. 4A). Although the combined therapy did not show a statistically significant difference over anti-4-1BB alone (p = 0.18), the addition of anti-4-1BB to the adoptive transfer model consistently increased the number of surviving mice compared to rat IgG control, in this and subsequent experiments. Of note, all mice that received reactivated T cells and anti-4-1BB survived the initial tumor were also resistant to subsequent tumor challenge (supplementary Figure S1).

### Anti-4-1BB enhances the expression of markers of survival and CTL effector function in reactivated memory T cells

The T cells used for adoptive transfer in this study are highly activated with potent CTL function (Fig. 1B). Nevertheless, we asked whether anti-4-1BB could further enhance their activation. Figure 4B shows that reactivation of memory T cells with peptide and control antibody leads to maximal activation as measured by CD69, CD44 and 4-1BB expression, as there is no further enhancement with addition of anti-4-1BB to the cultures. In contrast, the addition of anti-4-1BB to the memory T cells during their reactivation leads to increases in the survival marker Bcl-x_L_ as well as increases in granzyme B, a marker of CTL function (Fig. 4C–E). Thus, remarkably, anti-4-1BB can further activate even these highly stimulated reactivated memory T cells. To further investigate whether anti-4-1BB-mediated enhanced effector function was specific to reactivated memory cells, naïve CD8 T cells were stimulated with Ag under same conditions in the presence of anti-4-1BB. Compared to the robust granzyme B induction with anti-4-1BB on reactivated memory cells, anti-4-1BB was found to have a minimal effect in enhancing granzyme B expression on primary effectors derived from naïve CD8 OT-I (Fig. 4F). This result adds further impetus to the decision to use effectors reactivated from memory T cells, rather than primary effectors for the combined therapy with anti-4-1BB.

### Anti-4-1BB therapy expands innate immune cell subsets as well as endogenous and transferred T cells

**Figure 5:**
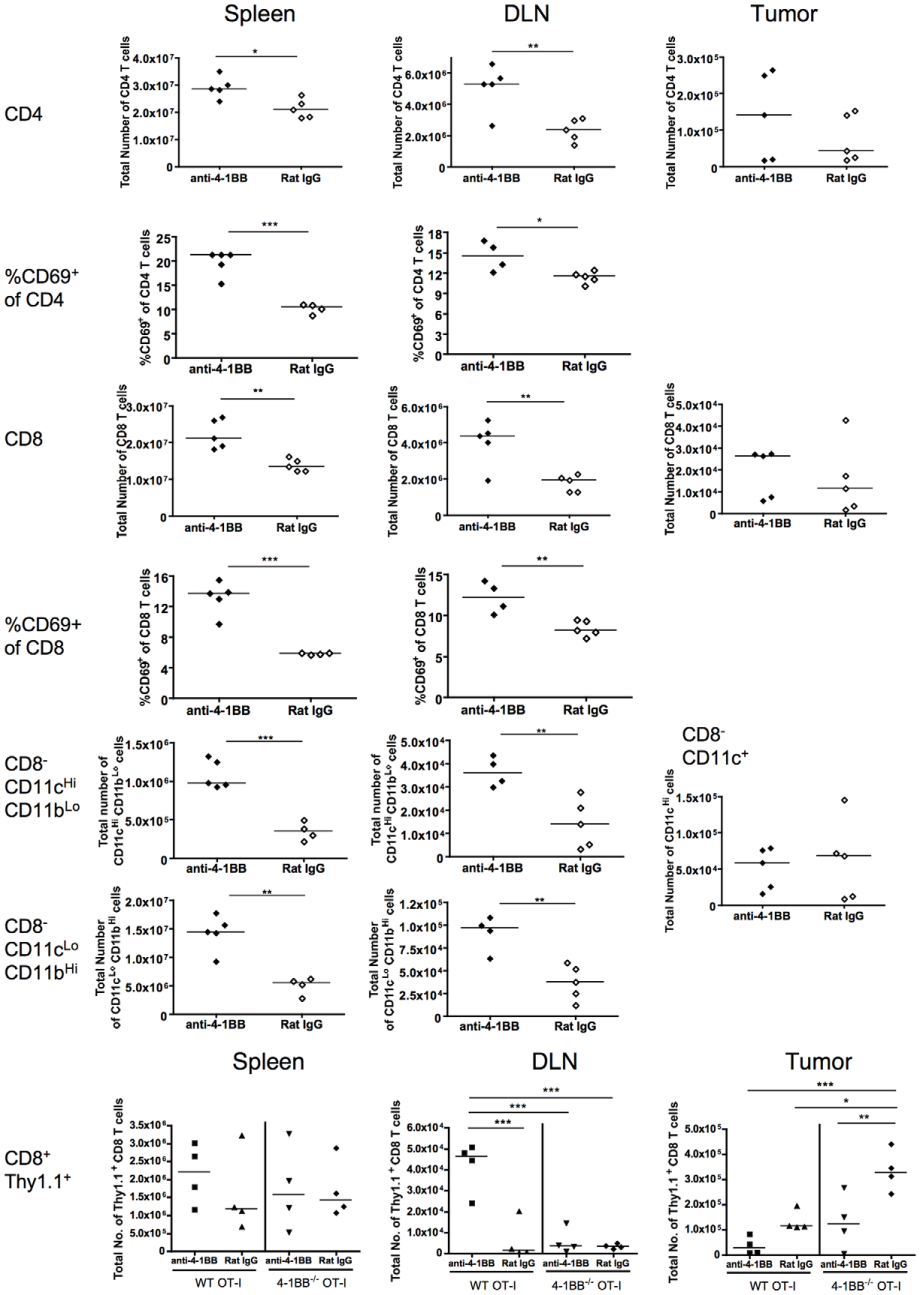
Effect of systemic anti-4-1BB on the frequency and number of immune cells recovered. Mice bearing 8-day E.G7 tumors were treated with reactivated memory OT-I T cells and anti-4-1BB or rat IgG control as in Figure 4A. On day 4, mice were sacrificed and the number of CD4, CD8 T cells, CD11c ^hi^ and CD11c^int^ CD8-negative cells (DC and macrophages), as well as transferred T cells were analyzed by flow cytometry. In the bottom panel, recovery of WT reactivated memory OT-I T cells was compared with the 4-1BB^−/−^ reactivated memory OT-I T cells, indicating that OT-I T cells can only expand in response to anti-4-1BB when they express 4-1BB. Each data point represents an individual mouse. Data are representative of three similar experiments. Results comparing two groups were analyzed by unpaired t-test, and one-way ANOVA was used for analysis of multiple groups in the bottom panel with *P<0.05, **P<0.01, and ***P<0.001.

A number of studies have used immunodepletion of different immune cell subsets to elucidate the cellular targets of 4-1BB in a tumor therapy setting. Depending on the particular model, CD8, CD4, DC and NK cells have all been implicated in anti-4-1BB therapies of cancer based on such immunodepletion studies prior to treatment [12], [24]–[29], [38]. Moreover, 4-1BB signaling has been shown to enhance dendritic cell and NK cell function [39]–[42]. While such experiments show that tumor control can require the interplay between different immune subsets, their complete deletion does not distinguish between anti-4-1BB agonists acting directly on these populations or a 4-1BB-independent requirement for these subsets in tumor control. Therefore, we evaluated the effect of anti-4-1BB on different immune subsets following adoptive therapy with OT-I T cells *in vivo*. The endogenous T cells were distinguished from transferred T cells by their lack of Thy1.1 expression. Compared to control rat IgG, anti-4-1BB increased the number of endogenous CD4 and CD8 T cells in the spleen and DLN of the mice. Moreover, transferred OT-I T cells also increased in number in the DLN upon anti-4-1BB treatment, but only if they expressed 4-1BB (Fig. 5, bottom panel). However, we did not see an increase in total T cell numbers in the tumor, perhaps because in the anti-4-1BB treated group, the tumor regresses rapidly and the T cells are no longer retained. In addition to effects on T cells, anti-4-1BB also increased the number of CD11c^hi^ and CD11c^int^ CD8-negative populations in spleen and DLN, indicating an effect on host dendritic cells and macrophages (Fig. 5). Again, significant effects of anti-4-1BB on the cells infiltrating the tumor were not seen. We also examined NK1.1^+^CD8^−^CD3^+^ and NK1.1^+^CD3^−^ cells, but the enhancement of NK1.1^+^ cell numbers was seen in only one of three experiments (data not shown).

### 4-1BB on the transferred T cells or on host cells is sufficient for the anti-tumor effect of anti-4-1BB

**Figure 6:**
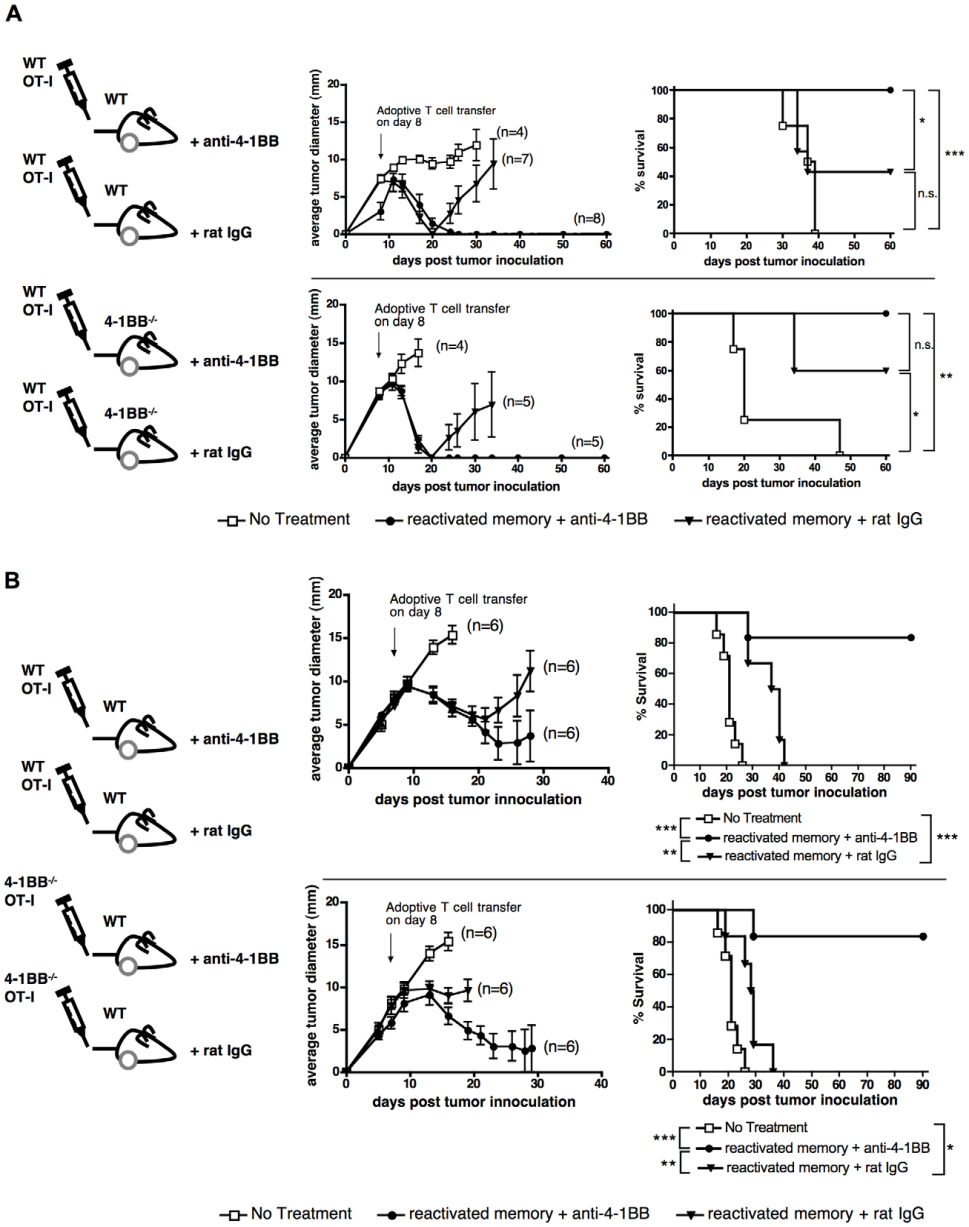
4-1BB on either transferred T cells or host cells is sufficient for the therapeutic effect of anti-4-1BB. (A) E.G7 tumors were allowed to grow for 8 days in WT mice or 4-1BB-deficient mice and then inoculated with 3×10^6^ WT reactivated memory cells followed by two doses of anti-4-1BB or rat IgG treatment. Average tumor growth and survival of tumor bearing mice is shown, where n indicates the number of mice per group. Results obtained with another set of 4-1BB^−/−^ mice showed a similar trend (data not shown). (B) WT mice bearing 8 day-tumors were treated with WT or 4-1BB^−/−^ reactivated OT-I memory cells followed by two doses of anti-4-1BB or rat IgG. Average tumor growth and survival curves of the tumor bearing mice are shown. Survival curves were analyzed by the LogRank test, with *P<0.05, **P<0.01 and ***P<0.001.

The above data indicated that anti-4-1BB therapy increases the numbers of innate immune cells, such as dendritic cells, as well as having effects on recovery of endogenous and transferred T cells. Thus it was of interest to determine which of these cell populations were important in the therapeutic response to anti-4-1BB. Therefore, we transferred reactivated memory cells together with anti-4-1BB into WT and 4-1BB-deficient tumor bearing hosts (Fig. 6A). The results show that 4-1BB in the host is completely dispensable for tumor control and mouse survival in response to the anti-4-1BB therapy in this combined adoptive T cell therapy model. Thus 4-1BB on the adoptively transferred T cells is sufficient to mediate the effect of anti-4-1BB in this adoptive immunotherapy model.

We next determined whether 4-1BB on the transferred T cells was important in the effect of anti-4-1BB on tumor control. When transferred T cells lacked 4-1BB, but host cells expressed 4-1BB, we still observed an enhancement of tumor clearance by anti-4-1BB (Fig. 6B). Thus, although 4-1BB is sufficient on the transferred T cells for the response to anti-4-1BB, it is not required when 4-1BB is present in the host (Fig. 6B). Although we did not detect 4-1BB expression on the E.G7 tumor (data not shown) and anti-4-1BB is not thought to be depleting, to rule out a possible direct role of anti-4-1BB on tumor growth, we conducted a control experiment in which 4-1BB-deficient mice were implanted with the E.G7 tumor and then treated with anti-4-1BB on day 8 and day 11 post-tumor implantation, without any T cell transfer. Under these conditions, tumors progressed in all groups (supplementary Figure S2). Thus anti-4-1BB has no direct effect on the tumor and anti-4-1BB therapy can be effective if either the transferred T cells or the host cells express 4-1BB, implying that 4-1BB on the transferred T cells or on host cells is sufficient for anti-4-1BB to contribute to tumor control in the adoptive immunotherapy model. The finding that anti-4-1BB was indeed effective when transferred cells lack but host cells express 4-1BB, also explains the substantial effect of anti-4-1BB in tumor control even in the absence of transferred T cells (Figure 4A).

Even though 4-1BB in the host was sufficient for the anti-4-1BB therapy, when OT-I T cells lacked 4-1BB, they showed decreased tumor control in the rat IgG control group compared to WT OT-I T cells (Fig. 6B, p = 0.03) suggesting that endogenous 4-1BBL can contribute to the therapy through effects on the transferred T cells. However, systemic addition of anti-4-1BB, through its effects in the host, can compensate for the lack of 4-1BB on the transferred T cells.

### On which cells in the host is 4-1BB required for anti-4-1BB induced therapy?

**Figure 7:**
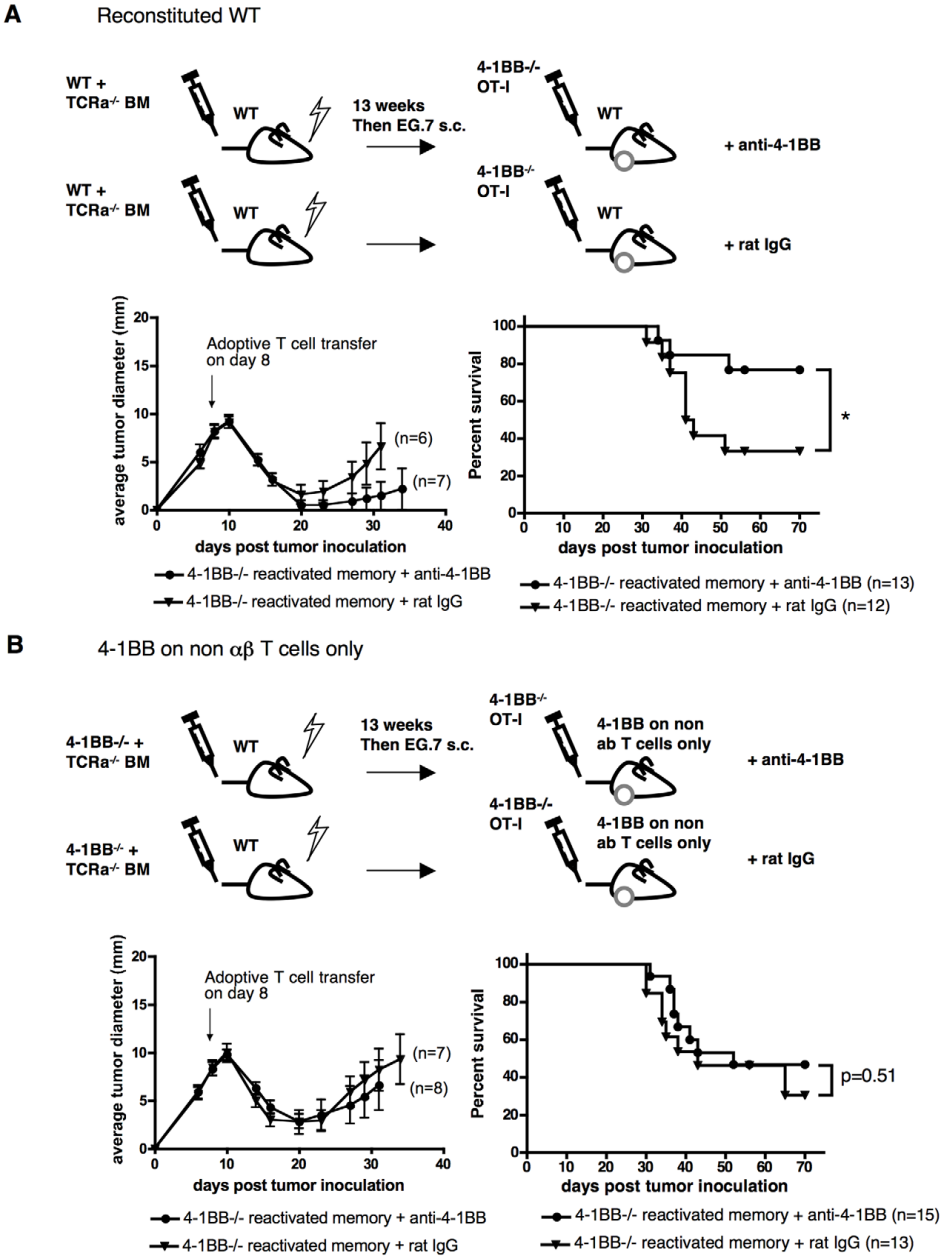
4-1BB on non-αβ T cells in the host is insufficient for anti-4-1BB mediated tumor control. Mixed bone marrow chimeras were generated by reconstituting lethally irradiated C57BL/6 WT mice with a 4∶1 mixture of TCRα− deficient: WT or 4-1BB^−/−^ bone marrow cells as illustrated. 13 weeks post reconstitution, chimeric mice were inoculated with E.G7 cells s.c. and 8 days later were treated with reactivated 4-1BB^−/−^ OT-I, followed by two doses of anti-4-1BB or rat IgG treatments as in Figure 6B. Average tumor growth and survival are shown (n = number of mice per group). Tumor growth curves are representative of two independent experiments with 6–7 mice per group each. Survival curves include the combined survival data from the two independent experiments.

The above experiments suggest that 4-1BB on either the transferred T cells or on cells in the host is sufficient for anti-4-1BB-induced therapeutic effects in this adoptive immunotherapy model. As both T cells and innate cells in the host were expanded in response to anti-4-1BB treatment (Fig. 5), it was possible that several cell types were involved. To evaluate this possibility, we generated mixed bone marrow chimeras in which 4-1BB was only absent from host αβ T cells, but not from other cells in the host (Fig. 7). The results show that when the transferred T cells lack 4-1BB, anti-4-1BB dependent tumor therapy requires 4-1BB on host αβ T cells. Thus the effect of agonistic anti-4-1BB antibody on adoptive T cell therapy requires 4-1BB expression on either transferred or endogenous αβ T cells and 4-1BB on other cell types is dispensable for the effects of anti-4-1BB.

## Discussion

Previous studies have shown the greater efficacy of central memory over effector memory T cells in anti-cancer therapy, attributed to their greater longevity *in vivo* [19], [20]. On the other hand, effector cells derived from reactivation of central memory T cells show superior persistence compared to primary effectors *in vivo*, suggesting that the combined effects of the enhanced survival of memory T cells and the immediate cytotoxic activity of activated effectors might be combined as a therapy [23]. The present study supports the superiority of reactivated memory cells over resting central memory cells in antigen-specific tumor control in a pre-established E.G7 tumor model (Fig. 1, 2).

Analysis of T cell accumulation in the tumor, spleen and draining LN showed that although central memory T cells had an initial survival advantage, they rapidly disappeared, whereas the reactivated memory cells continued to accumulate. CFSE labeling experiments showed that although the resting memory cells were capable of division in the tumor and draining LN, they showed less division than their reactivated counterparts (Fig. 3). This suggested that antigen presentation *in vivo* was limiting, in agreement with previous studies showing that there is poor presentation of tumor antigens *in vivo* [43], [44]. The tumor microenvironment is known to be immunosuppressive and not conducive to strong antigen presentation [45]. Even though we used a model system in which a foreign antigen is introduced into the tumor and we transfer 3 million T cells that have high affinity for this antigen, unless we pre-activate the T cells there is relatively little activation of the T cells against the pre-established tumor. Thus, although the model may be contrived to allow us to readily follow the T cell response, it is clearly a stringent model with aggressive tumor growth in the absence of a highly optimized therapy.

The advantage of using reactivated memory cells, is that by giving them their antigen *ex vivo*, one allows the cells to commit to programmed expansion [37], even though antigen presentation in the tumors may be limited. The use of reactivated memory T cells of an irrelevant specificity confirmed this hypothesis. P14 TCR transgenic T cells activated *ex vivo*, continued to divide *in vivo* and accumulated at levels comparable to reactivated OT-I memory T cells, although, as expected, they fail to control the tumor. The failure of the adoptively transferred “resting” memory T cells to be effectively reactivated in the tumor bearing host also supports the idea that the use of *ex vivo* reactivated memory T cells allows tumor control, without a requirement for the T cells to be reactivated in the suppressive microenvironment of the tumor. These studies suggest that for human therapy, the expansion of tumor-infiltrating lymphocytes with Ag followed by IL-15 *in vitro*, and then reactivation with Ag before reinfusion will provide improved protection compared to using central memory T cells.

Recently, Klebanoff et al. came to a similar conclusion about the effectiveness of *in vitro* reactivation of memory CD8 T cells for cancer control [17]. In their study, effector memory T cells were reactivated and then infused into a sublethaly irradiated tumor-bearing host followed by systemic treatment of the mice with IL-2. Our studies extend this result to a non-irradiated mouse and also avoid the use of IL-2, both of which would independently contribute to further T cell expansion. The study of Klebanoff also showed that the pre-activation of the T cells allowed Ag-independent release of IFNγ, which in turn acted on tumor cells to induce MHC I, which the authors proposed was key to rendering the tumors susceptible to tumor killing. However, the antigen specificity of these reactivated effectors was not tested [17]. In the present study, we show that activated P14 T cells, while continuing to divide and enter the tumor, fail to have any impact on tumor growth. Thus by comparing P14 and OT-I T cells, we show that the property of the T cells to enter the tumor and divide is due to their *ex vivo* stimulation, whereas, the tumor killing is antigen specific and not due to T cell cytokine release alone.

Anti-4-1BB has shown promise in a number of anti-cancer therapeutic models (reviewed in [13], [14]). Consistent with a previous study on combined use of agonistic anti-4-1BB with adoptive T cell therapy [27], agonistic anti-4-1BB antibodies provided additional effects over and above the reactivated memory cells, largely preventing relapse and resulting in 80% cure of the 8-day pre-established tumor (Fig. 4A); in contrast, the rat IgG control had no effect over and above the T cells alone. It should be pointed out however, that the major effect in our study appears to be with the anti-4-1BB agonist antibody, which gave 60% tumor control when used alone. The combination with T cells plus anti-4-1BB showed a trend towards increased survival over anti-4-1BB alone (80% survival; p = 0.18). Although in our studies, it was sufficient for 4-1BB to act on the endogenous T cells (Fig. 6, 7), in a clinical setting, where the patient may be more immunosuppressed, the combination of anti-4-1BB with adoptive therapy may be more important as compared to monotherapy with anti-4-1BB alone.

The mechanism by which 4-1BB improves tumor control is likely due to a combination of its ability to increase T cell survival/expansion, as well as the ability to increase the CTL function of the transferred and/or responding host T cells (Fig. 4C–E and Fig. 5). In the absence of anti-4-1BB therapy, it was clear that the reactivated memory T cells were enriched in the tumor at day 4 compared to the resting memory T cells (Fig. 3) and had a more activated phenotype than the resting memory T cells or the reactivated memory T cells that had gone to the spleen or draining LN (Fig. 3B). Paradoxically, however, while anti-4-1BB increased the number of OT-I T cells in the spleen and draining LN, we did not observe an increase in OT-I or host T cells in the tumor after anti-4-1BB therapy. It is worth noting that Ag-non-specific P14 T cells accumulated at higher numbers in the tumor than the tumor specific OT-I T cells (Fig. 3C). Therefore, it is possible that the rapid regression of the tumors upon anti-4-1BB and adoptive T cell therapy resulted in lower recovery of the T cells extracted for analysis in the case of Ag-specific as compared to Ag non-specific T cells. The finding that the Ag non-specific P14 cells are enriched in the tumor to apparently higher levels than the Ag-specific T cells, argues that the presence of T cells in the tumor is not a good correlate of protection for the combined anti-4-1BB/adoptive T cell therapy.

It was recently reported that effectors derived from naïve T cells are superior in tumor control compared to the effectors that were derived from central memory CD8 T cells, with apparently enhanced proliferative potential, enhanced cytokine production, as well as the lack of KLRG-1, a marker for proliferative senescence [46]. Notwithstanding the difficulty of obtaining primary effectors in large quantities from tumor infiltrating lymphocytes, the present study shows that anti-4-1BB has a much more substantial effect on enhancing granzyme B expression in reactivated memory T cells as compared to its minimal effect on primary effectors (Fig. 4F).

A recent study determined that for elimination of solid tumors in mice, it is the density of T cells (the critical T cell concentration, or CTC) in the tumor rather than the overall effector to target ratio that determines whether the tumor relapses or a cure is achieved [47]. Moreover, the authors calculated that the CTC must be maintained for at least 8 days to affect a cure. In this regard, it is of interest that treatment with reactivated memory T cells alone resulted in initial regression of the tumors, followed by a relapse a few days later. In contrast, the combination of anti-4-1BB and the reactivated memory T cells resulted in the majority of animals achieving a cure. Moreover, it was necessary and sufficient for either the transferred T cells or the endogenous T cells to express 4-1BB for this effect. Thus, the ability of anti-4-1BB antibodies to extend the lifetime of either endogenous responding effectors or transferred T cells, may be important in maintaining the critical T cell concentration for a cure. However, as noted above, it is difficult to confirm the density of the CD8 T cells in the tumor over time experimentally, as the tumors rapidly regress.

Melero et al. [12] first demonstrated the use of agonistic anti-4-1BB antibodies as a cancer therapy and this approach has now moved to phase I clinical trials for advanced cancers **(** http://www.clinicaltrials.gov keyword CD137). Subsequent work has shown that in some models, not only are CD4 and CD8 T cells targeted by anti-4-1BB, but that the effect of anti-4-1BB requires the presence of NK cells [24], [26], [48]. The enhancement of NK cell cytokine production by anti-4-1BB can in turn enhance dendritic cell function [25], [27], [49]. Moreover, recent studies show that depletion of DC results in lack of efficacy of anti-4-1BB therapy [28]. It is clear that T cells modified to express chimeric 4-1BB signaling molecules can control tumors, arguing for direct effects of 4-1BB on CD8 T cells in cancer therapy (for example, [50]–[52]). However, it was unclear in the present model, whether anti-4-1BB was acting directly on the transferred OT-I T cells or on the host immune system or both. The results presented here show that although anti-4-1BB expands a number of host cell subsets, including T cells, dendritic cells and macrophages, this function of 4-1BB is dispensable for the therapeutic effect when the transferred T cells express 4-1BB. On the other hand, the reciprocal experiment in which the host cells express 4-1BB, but transferred T cells do not, also allowed anti-4-1BB to increase the effectiveness of the adoptive immunotherapy, indicating that either host cells or adoptively transferred T cells were necessary and sufficient for the effect of agonist anti-4-1BB on tumor growth. Further experiments showed that it was the host αβ T cells and not other 4-1BB expressing subsets (such as γδ T cells, NK cells, or dendritic cells) that are required for effects of anti-4-1BB on tumor control in this model.

Recently, Narazaki et al. [53] showed in a model in which transferred T cells are used to prevent tumor recurrence in a rechallenge model, that for agonistic anti-4-1BB therapy, 4-1BB was more important on the transferred T cells with lesser effects on host cells. They went on to show that 4-1BB on radioresistant cells was contributing to this minor effect. They suggested this could be an NK cell or DC, but this is highly unlikely as these cells are radiosensitive. Rather, it is possible that their observations were due to incomplete elimination of memory T cells, which are highly responsive to 4-1BB agonists [9] and more difficult to replace in a radiation chimera. Our study, in contrast, clearly shows that when transferred T cells lack 4-1BB, the absence of 4-1BB on host αβ T cells results in loss of the therapeutic effect of anti-4-1BB agonist. Thus although 4-1BB agonists can expand many cell types, it is T cells that are critical for the therapeutic effect, at least in this model. This finding is consistent with a previous report that the enhancement of T cell memory by anti-4-1BB treatment during immunization also requires T cell intrinsic 4-1BB [54].

Agonistic anti-4-1BB antibodies are highly potent in expanding both memory T cells as well as innate immune subsets in mice. This has been associated with splenomegaly, and other immune system and hematopoietic anomalies observed at doses starting at 200 µg of anti-4-1BB per mouse [30]. In our model, we used two doses of 100 µg of the anti-4-1BB antibody per mouse and found that this was sufficient to allow 80% mouse survival when combined with adoptive T cell transfer. Moreover, 8/8 mice that survived their tumors after anti-4-1BB and adoptive T cell therapy were resistant to a second tumor challenge at day 66 and remained healthy for at least another 60 days after initial tumor control. Therefore, it appears that two low doses of anti-4-1BB were well tolerated with no appearance of obvious side effects for at least 120 days post-treatment. On the other hand, in a recent study, delivery of three consecutive weekly doses of 150 µg of a different anti-4-1BB agonistic antibody (clone 1D8 or 2A) to mice resulted in significant T cell mediated liver inflammation and immunopathology, without control of a murine colon carcinoma [55]. Thus the therapeutic window for 4-1BB to enhance T cell mediated tumor control, while avoiding T cell-mediated toxicity appears to be quite narrow. In this regard, it is of interest that alternate forms of 4-1BB stimulation of T cells may be less toxic than systemic administration of antibodies [56]–[58]. It might also be possible to use anti-4-1BB *ex vivo* to enhance the survival and effector function of the T cells prior to transfer. However, in our hands, pilot experiments in which we used anti-4-1BB during the reactivation of the memory T cells *ex vivo* did not result in the same therapeutic effect as obtained with *in vivo* administration of anti-4-1BB. Thus, this approach will likely require some optimization.

In conclusion, the results of this study extend previous studies suggesting that for optimal CD8 T cell adoptive therapy, it is more efficacious to reactivate memory T cells before infusion, rather than relying on *in vivo* antigen presentation. Our studies show that this in turn allows these effectors to continue to divide *in vivo* independently of the presence of antigen, even in the suppressive microenvironment of the tumor. Reactivated memory T cells in combination with anti-4-1BB agonist therapy represent an even more powerful combination for immunotherapy of cancer. Remarkably our studies show that anti-4-1BB can further activate even highly activated effectors T cells derived from central memory T cells. We further show that the major effect of anti-4-1BB is on the T cells and that it is necessary and sufficient for either the adoptively transferred CD8 T cells or the host αβ T cells to express 4-1BB for the effects of anti-4-1BB to be realized. Conversely, although other 4-1BB-expressing immune cell subsets may contribute to the anti-tumor immune response, they do not need to express 4-1BB for anti-4-1BB to exert its effects in this model. As therapy with anti-4-1BB moves forward in the clinic, it becomes more important to understand the way in which anti-4-1BB is acting, in order to rationally design improvements and combination therapies [15].

## Materials and Methods

### Mice

C57BL/6 wild type mice were purchased from Charles River Laboratories (St. Constant, QC, Canada). 4-1BB^−/−^ mice extensively backcrossed to the C57BL/6 background have been described [59]. OT-I mice [60], CD45.1 and Thy1.1 congenic mice were obtained from Jackson Laboratories (Bar Harbor, Maine, USA) and crossed to generate CD45.1 OT-I, Thy1.1 OT-I, and Thy1.1 4-1BB^−/−^ OT-I mice. OT-I transgene was detected using anti-Vα2 and anti-Vβ5.1/5.2 antibodies (eBioscience, San Diego, CA). Mice were maintained under SPF conditions in sterile microisolators.

### Ethics statement

All animal studies were approved by the University of Toronto animal care committee in accordance with the regulations of the Canadian Council on animal care (University of Toronto approved protocol #20007828).

### Generation of memory T cells *in vitro*

CD8 T cells with a central memory phenotype were generated by culture with Ag followed by IL-15 [61], [62] with modifications as described [6]. In brief, OT-I splenocytes were stimulated with SIINFEKL peptide (0.1 µg/ml) for 2 days, then the non-adherent cells were rested for 1 day in fresh media (RPMI-1640 with 10% heat-inactivated fetal calf serum, 0.03% L-glutamine, antibiotics and 2-mercaptoethanol) then cultured in media containing 20 ng/ml recombinant human IL-15 (R&D, Minneapolis, MN). Media was replaced every 2 days for a total culture time of 9 days, by which time more than 95% of the viable cells were CD8^+^, Vα2^+^ and Vβ5^+^. To reactivate memory cells, irradiated (2000 rads), SIINFEKL peptide loaded C57BL/6 splenocytes were added to the memory cell cultures with IL-15 for the last 2 days at a ratio of one feeder cell to one T cell. Viable cells were collected by lympholyte gradient centrifugation (Cedarlane, Hornby, ON, Canada). For carboxyfluorescein diacetate succinimidyl ester (CFSE, Molecular Probes, Eugene, OR) labelling, 10^7^/ml T cells were incubated in pre-warmed PBS/2%FCS containing 5 µM CFSE for 15 minutes at 37°C followed by 5 minutes quench on ice, extensive washing and resuspension in PBS for adoptive transfer. Activated memory T cells with an irrelevant Ag specificity were generated using P14 transgenic mice [34], using the same protocol, except that the LCMV glycoprotein peptide epitope gp_33–41_ [34] was used.

### Analysis of T cell cytotoxicity

Viable resting or reactivated memory OT-I cells were analyzed for the cytotoxic activity against ^51^Cr-labeled E.G7 or control EL-4 target cells in a standard 4 hr ^51^Cr release assay, as described [63]. The percentage of specific lysis was calculated using the following equation: (experimental ^51^Cr release–spontaneous ^51^Cr release)/(maximum ^51^Cr release–spontaneous ^51^Cr release)×100% = %specific lysis.

### The E.G7 tumor model and immunotherapy of tumor bearing mice

The E.G7 murine thymoma cells (American Type Culture Collection, Manassas, VA) were freshly thawed and maintained in media containing 0.4 mg/ml Geneticin (Invitrogen, Carlsbad, CA). Six to ten week old mice were implanted subcutaneously on the right flank with 5×10^5^ or 2×10^6^ E.G7 cells in PBS. Tumor growth was monitored by measuring two perpendicular diameters of the tumor using calipers. For adoptive transfer, a total of 3×10^6^ T cells in 0.2 ml PBS were injected via the tail vein into tumor bearing mice. Where indicated, anti-4-1BB antibody (clone 3H3 [7], kindly provided by Dr. Robert Mittler, Emory University) or the rat IgG control was administered intraperitoneally at a dose of 100 µg in 100 µl, immediately after T cell adoptive transfer and again after 3 days.

### Flow cytometry

Phenotyping of OT-I cells was carried out by surface staining with fluorescent anti-mouse CD8α, CD44, CD62L, CD69, 4-1BB antibodies or isotype controls (eBioscience). Single cell suspensions of the tumor, draining lymph node (right inguinal) and spleen were analyzed at the indicated times post-transfer. Tumor tissue was digested with 1 mg/ml collagenase D (Roche, Indianapolis, IN), 0.1 mg/ml hyaluronidase, type V (Sigma) and 20 unit/ml DNase, type IV (Sigma) for 1.5 hours at room temperature. OT-I cells were identified using the Thy1.1 congenic maker. For detection of endogenous immune subsets, cell suspensions were first blocked with anti-FcR (eBioscience), followed by surface staining with anti-mouse CD11c, CD11b, CD3, CD4, CD8, CD69 (eBioscience) and NK1.1 (BD bioscience, Mountain View, CA). For intracellular IFN-γ staining, cells were restimulated with SIINFEKL for 4.5–5 hours at 37°C in the presence of Golgi stop (BD Biosciences). Cells were surface-stained with Cy-chrome-anti-CD8 and APC-anti-Thy1.1 antibodies, fixed, permeabilized and stained with PE-anti-mouse IFN-γ antibody or the isotype control (BD Biosciences). For detection of Bcl-x_L_ and granzyme B following *in vitro* reactivation, viable cells were collected by lympholyte gradient centrifugation (Cedarlane), then live cells were fixed and permeabilized (BD Bioscience), followed by intracellular staining with anti-Bcl-x_L_ (SouthernBiotech, Birmingham, AL) and anti-granzyme B antibodies (ebiosence). Data were acquired on a FACSCalibur with the CellQuest software (BD Biosciences,) and analyzed using FlowJo software (TreeStar Inc., CA).

### Bone marrow chimeras

Bone marrow cells were obtained from femurs and tibia of WT, TCRα^−/−^, and 4-1BB^−/−^ mice. C57BL/6 mice were lethally irradiated with two doses of 550 rads, followed by reconstitution with a 4∶1 ratio of TCRα^−/−^ to WT or 4-1BB^−/−^ bone marrow cells through intravenous injection at a total number of 5×10^6^ cells in 200 µL volume. The 4∶1 ratio was needed to ensure that the majority of the non-T cells would be competent in expressing 4-1BB in the 4-1BB^−/−^ TCRα^−/−^ mixed bone marrow chimeras. Reconstituted mice were given water supplemented with 2 mg/mL of neomycin sulfate (Bio-Shop, Burlington, Ontario, Canada) during the first four weeks, and they were further rested for additional 2 months before tumor inoculation.

## Supporting Information

Figure S1 Mice that received anti-4-1BB and survived the initial tumor were fully protected against subsequent tumor challenge. Tumor free WT survivors from the group treated with WT reactivated OT-I followed by anti-4-1BB or rat IgG treatment from the experiment shown in 6A were rechallenged with 2×106 E.G7 tumor cells s.c. on day 60 post primary tumor inoculation without further treatment. Average tumor growth and survival of the naïve and rechallenged mice are shown on the left and right panel, respectively. (n) indicates the number of mice in each group. Survival curves were analyzed by the LogRank test, with *P<0.05, and ***P<0.001.
(0.16 MB TIF)

Figure S2 Lack of control of E.G7 tumor following anti-4-1BB treatment in 4-1BB-deficient mice. 4-1BB-/- mice were inoculated with E.G7 tumor cells on day 0, followed by two injections of anti-4-1BB or rat IgG on day 8 and 11. Average tumor growth and survival curves are shown on the left and right panel, respectively. Data represent one experiment with 6 mice per group.
(0.16 MB TIF)
