# Supplementary figures and images for: Evaluating the Cellular Targets of Anti-4-1BB Agonist Antibody during Immunotherapy of a Pre-Established Tumor in Mice
Source: PLoS One. 2010 Jun 8;5(6):e11003. doi: 10.1371/journal.pone.0011003 (PMC2882368; doi:10.1371/journal.pone.0011003)

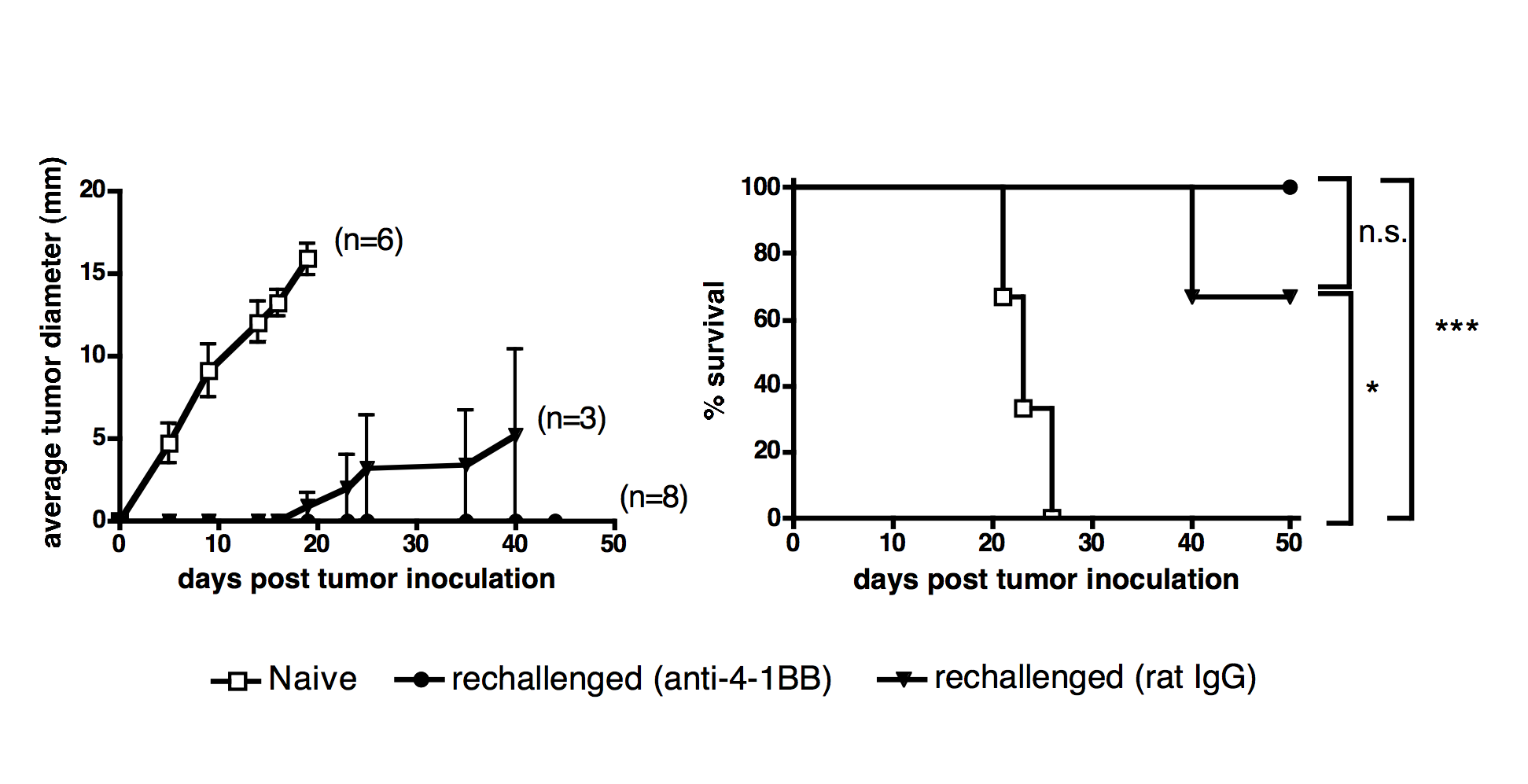

Supplement: Figure S1 — Mice that received anti-4-1BB and survived the initial tumor were fully protected against subsequent tumor challenge. Tumor free WT survivors from the group treated with WT reactivated OT-I followed by anti-4-1BB or rat IgG treatment from the experiment shown in 6A were rechallenged with 2×10^6^ E.G7 tumor cells s.c. on day 60 post primary tumor inoculation without further treatment. Average tumor growth and survival of the naïve and rechallenged mice are shown on the left and right panel, respectively. (n) indicates the number of mice in each group. Survival curves were analyzed by the LogRank test, with *P<0.05, and ***P<0.001. (0.16 MB TIF) [file pone.0011003.s001.tif]

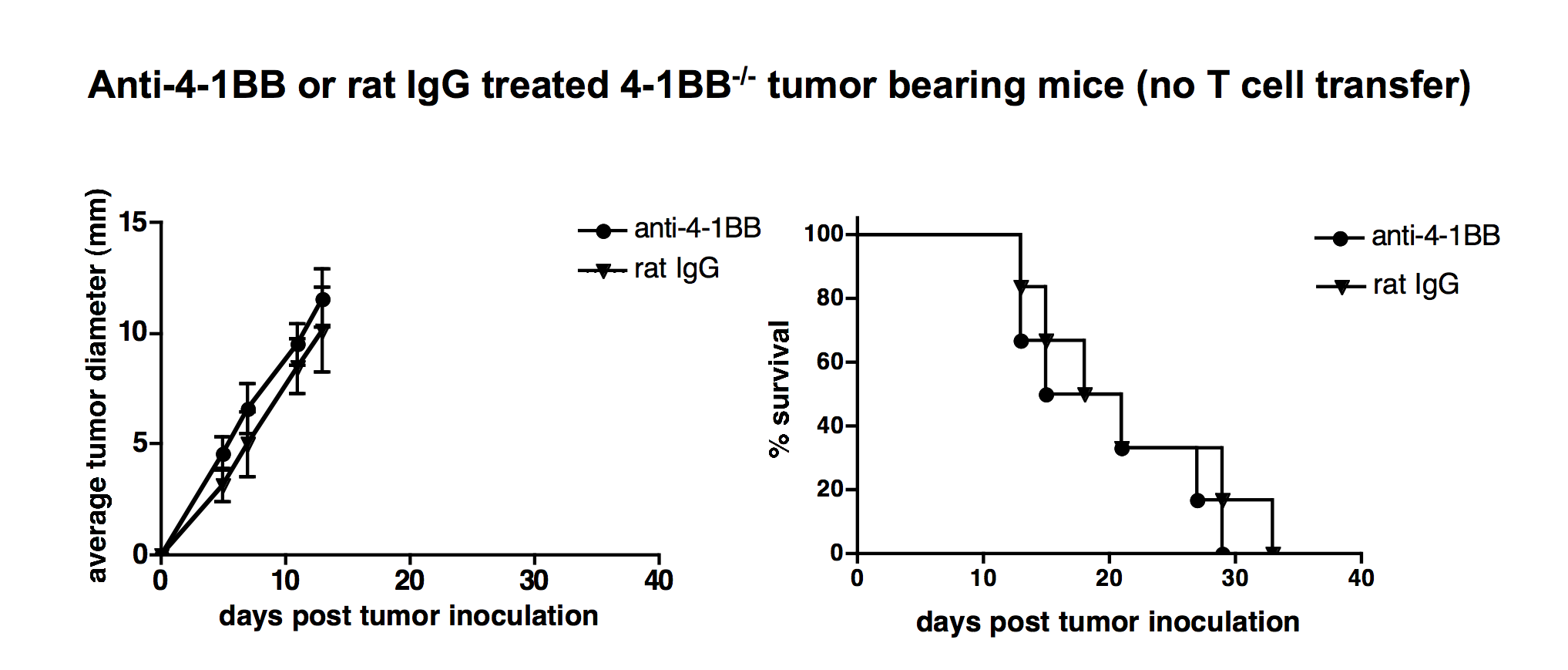

Supplement: Figure S2 — Lack of control of E.G7 tumor following anti-4-1BB treatment in 4-1BB-deficient mice. 4-1BB-/- mice were inoculated with E.G7 tumor cells on day 0, followed by two injections of anti-4-1BB or rat IgG on day 8 and 11. Average tumor growth and survival curves are shown on the left and right panel, respectively. Data represent one experiment with 6 mice per group. (0.16 MB TIF) [file pone.0011003.s002.tif]
